# Analysis of Prognostic Alternative Splicing Reveals the Landscape of Immune Microenvironment in Thyroid Cancer

**DOI:** 10.3389/fonc.2021.763886

**Published:** 2021-10-18

**Authors:** Jian Wu, Yifang Sun, Junzheng Li, Maomao Ai, Lihua You, Jianbo Shi, Feng Yu

**Affiliations:** ^1^ Department of Otorhinolaryngology-Head and Neck Surgery, Guangzhou Red Cross Hospital, Jinan University, Guangzhou, China; ^2^ Department of Ophthalmology, Guangzhou Red Cross Hospital, Jinan University, Guangzhou, China; ^3^ Department of Otorhinolaryngology, The First Affiliated Hospital of Sun Yat-Sen University, Sun Yat-Sen University, Guangzhou, China

**Keywords:** thyroid cancer, alternative splicing, immune microenvironment, immunotherapy, immune checkpoint (ICP)

## Abstract

**Background:**

The incidence of thyroid cancer (THCA) continues to increase in recent decades. Accumulating evidence showed that the unbalanced alternative splicing (AS) promotes the occurrence of cancers and leads to poor prognosis of patients. However, the research on alternative splicing events in THCA is lacking, and its underlying mechanism is not fully understood. This study identifies a novel prognostic signature based on AS events to reveal the relationship of AS with tumor immune microenvironment.

**Methods:**

Based on the AS data, transcriptional data, and clinical information, the differentially expressed alternative splicings (DEASs) were screened out. Least absolute shrinkage and selection operator (LASSO) regression and multi-Cox regression analyses were employed to identify prognostic results related to AS events and establish a prognostic signature. The predictive ability of the signature was assessed by Kaplan-Meier (K-M) survival curve, risk plots, and receiver operating characteristic (ROC) curves. Furthermore, correlations between tumor-infiltrating immune cells, immune checkpoints, immune score and prognostic signature were analyzed.

**Results:**

According to the LASSO regression analysis, a total of five AS events were selected to construct the signature. K-M survival curve showed that the higher the risk score, the worse the OS of the patients. Risk plots further confirmed this result. ROC curves indicated the high predictive efficiency of the prognostic signature. As for tumor immune microenvironment, patients in the high-risk group had a higher proportion of immune cells, including plasma cell, CD8+ T cell, macrophages (M0 and M2), and activated dendritic cell. Immune checkpoint proteins, such as PDCD1LG2, HAVCR2, CD274, etc., were significantly higher in the high-risk group. We also found that the ESTIMATE score, stromal score, and immune score were lower in the high-risk group, while the result of tumor purity was the opposite.

**Conclusions:**

Collectively, a prognostic signature consisting of five AS events in THCA was established. Furthermore, there was an inextricable correlation between immune cell infiltration, immune checkpoint proteins, and AS events. This study will provide a basis for THCA immunotherapy in the future.

## Introduction

The incidence of THCA continues to increase worldwide, mainly due to the progress of imaging technology and the increase of examination methods. The number of thyroid cancer cases detected in the USA each year increased by 240% from 1973 to 2002, eventually reaching 7.7 per 100,000 people. However, the data continues to increase, reaching 15.2 per 100,000 people in 2013 (1). The mechanisms of THCA are complex because they are controlled by genetic alterations in gene mutation, increased copy number of genes and abnormal methylation of genes, which lead to heterogeneity of the disease (2, 3). The challenge for clinical doctors in the treatment of thyroid cancer is to balance the treatments so that patients with low-risk disease or benign thyroid nodules are not overtreated. At the same time, doctors need to find new treatments as the traditional antineoplastic therapy did not achieve satisfactory results for all thyroid cancers (4). Therefore, there is an urgent need to explore new biological indicators and molecular mechanisms of THCA to help achieve accurate treatment and provide new targets for immunotherapy.

Gene sequencing technology, especially the next-generation sequencing, has developed rapidly in recent decades. It has become a trend to utilize big data of tumor genomics to excavate and analyze the internal factors affecting tumor formation and progression. Alternative splicing (AS) is one of the most important posttranscriptional regulation, which can modify more than 90% of human genes (5). Accumulating evidence showed that AS is widely involved in the formation of tumor microenvironment (6–9). Although there were some studies on the AS events in THCA (10, 11), the role of AS events in tumor immune microenvironment is lacking. Therefore, it is an unmet need to explore the potential mechanism of AS events on immune microenvironment in THCA.

In this research, we established a prediction model including five AS events on the basis of comprehensive bioinformatics analysis. ROC curve and K-M survival curve revealed the prediction capability of the prediction model in THCA. To reveal the underlying mechanism between AS events and 22 types of immune cells, CiberSort algorithm was employed to calculate the proportion of tumor immune cell infiltration between patients in the high- and low-risk groups. Finally, the potential immune checkpoints of patients were analyzed, which may help to break the bottleneck of THCA immunotherapy.

## Methods

### Data Collection

Transcriptional data and clinical information of 506 THCA patients were downloaded from The Cancer Genome Atlas (TCGA) database (https://tcga-data.nci.nih.gov/). AS data of THCA patients were downloaded from the SpliceSeq database (http://bioinformatics.mdanderson.org). Finally, a total of 495 THCA patients with splicing data, transcriptional data, and clinical information were included in this study for analysis.

### Identification of Survival-Related AS Events and Prognostic Signature Construction

For TCGA spliceseq, seven types of AS events were quantified by percent spliced in (PSI), which ranges from 0 to 1 (12). A PSI value ≥0.75 was selected as filter of all samples. Visualization of AS events was performed through the Upset plot, which was drawn using the UpSetR package (R software 4.0.5). Univariate Cox regression (set *p* < 0.05 as filter) was selected to identify the differentially expressed alternative splicings (DESAs), and the DEASs related to prognosis were selected for further analysis. The final survival-related AS events was identified by least absolute shrinkage and selection operator (LASSO) regression, which can avoid overfitting of model. Furthermore, multivariate Cox regression was employed to construct the prognostic signature model based on selected AS events. $\text{Risk score}=\sum_{i=0}^{n}=\text{PSI}*\text{coe}{\text{f}}_{i}$ , in which *n* represented the number of AS events selected by prognostic signature and coef*_i_* represented regression coefficient of each selected AS event.

### Validation of Prognostic Signature

According to the results of median risk score, 495 THCA patients were divided into the high- and low-risk groups. For K-M survival curve, the difference of overall survival time (OS) in the high- and low-risk groups was compared. The predictive efficiency of prognostic signature was evaluated by using ROC curve to calculate the survival rate of 1, 3, and 5 years. The hazard ratio (HR) of the risk score and clinical parameter were obtained by univariate and multivariate Cox regression analyses.

### Immune Cell Infiltration and Immune Checkpoint Analysis

CiberSort, a tool that can provide specific immune cell types based on RNA profile, was used to analyze the 22 immune cell subtypes in THCA. The vioplot package of R was used to identify different immune cell infiltration between the high- and low-risk groups. At the same time, the immune score was calculated according to the ESTIMATE algorithm. The ggpubr package of R was used to analyze the difference of immune features (stromal score, immune score, ESTIMATE, and tumor purity). Immune status and potential immunotherapy were predicted by comparing the differential expression of immune checkpoints between the high- and low-risk groups. Meanwhile, the correlation of immune checkpoints and the risk score was analyzed.

### Splicing Factors and DEAS Regulatory Network

AS-related genes were acquired from MSigDB, which was the abbreviation of the Molecular Signatures Database (13). The correlation between the PSI values of DEASs and expression of splicing factors (SFs) was assessed by the Pearson correlation analysis. Both correlation coefficient >0.6 and adjusted *p* < 0.001 were set as filtering conditions. Cytoscape (version 3.8.2) was used to visualize networks related to DEASs and splicing genes (14).

## Results

### Characteristics and AS Events of Patients With THCA

**Figure 1:**
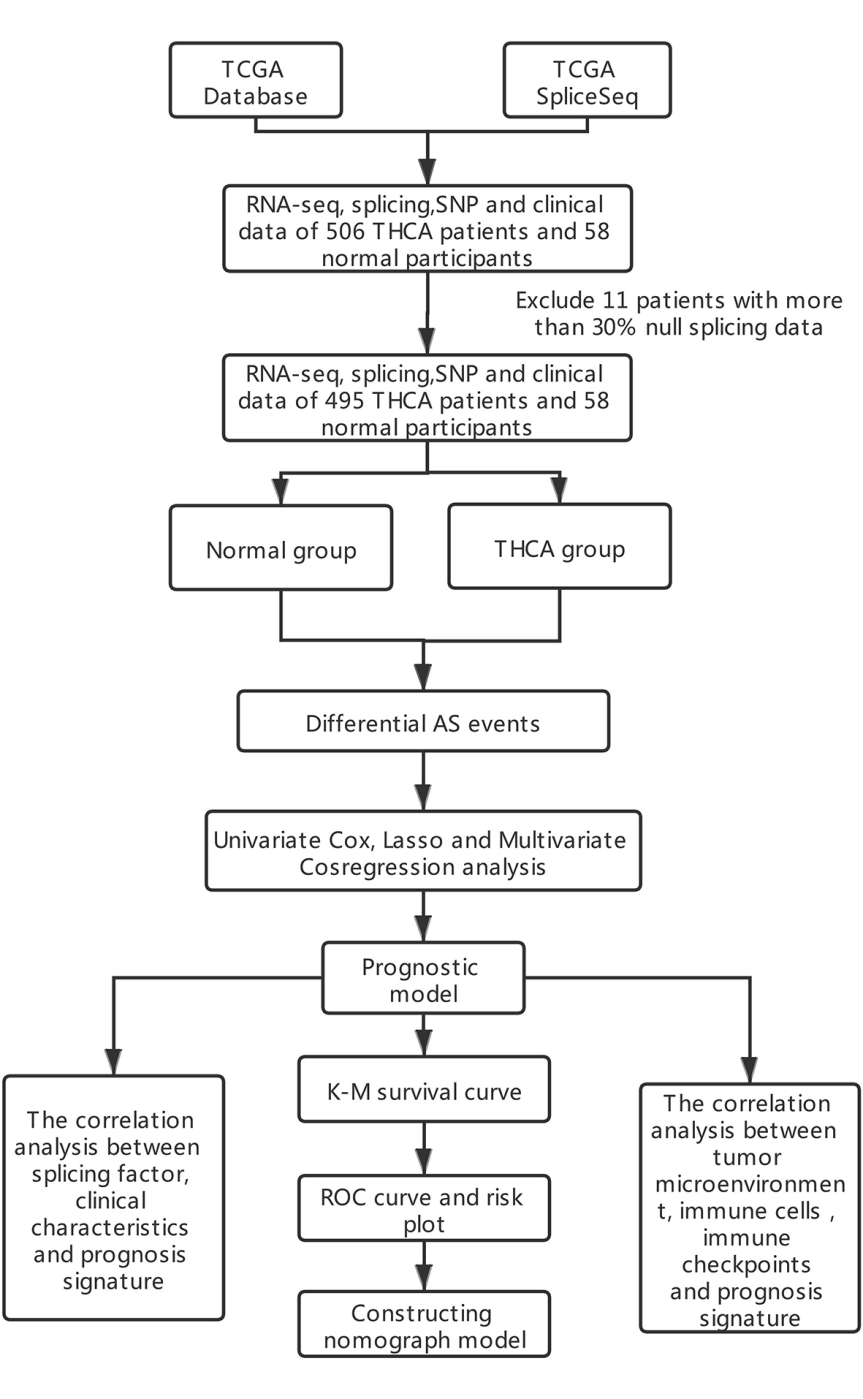
Flow diagram of this study.

**Figure 2:**
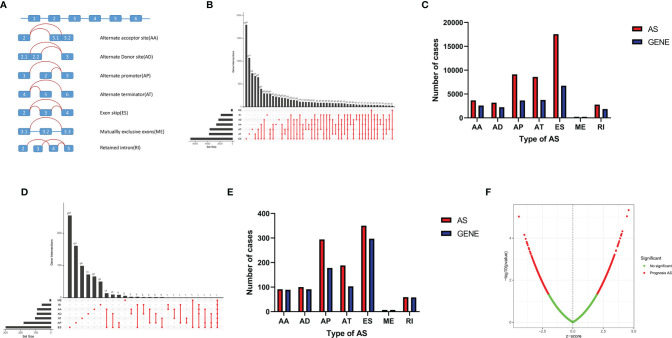
AS events in THCA. **(A)** Seven AS events types. **(B)** Upset plot of seven types of AS events and related genes in THCA. **(C)** Bar plot of AS events and related gene numbers in THCA. **(D)** Upset plot of OS-related AS events and related genes. **(E)** Bar plot of OS-related AS events and related genes numbers. **(F)** Volcano plot of prognostic AS events.

A total of 506 THCA patients from TCGA database were enrolled in the study, and 11 of them were excluded for null splicing data, which were over 30%. Details of the study are shown in the workflow (**Figure 1**). The clinical information of enrolled patients are displayed in **Table 1**. **Figure 2A** shows seven types of AS events, such as, the alternate acceptor site (AA), alternate donor site (AD), alternate promoter (AP), alternate terminator (AT), exon skip (ES), mutually exclusive exons (ME), and retained intron (RI). A total of 3,638 AA in 2,592 genes, 3,190 AD in 2,240 genes, 9,127 AP in 3,650 genes, 8,595 AT in 3,753 genes, 17,536 ES in 6,748 genes, 232 ME in 224 genes, and 2,787 RI in 1,865 genes were identified (**Figures 2B, C**).

**Table 1:** Characteristics of patients with THCA from TCGA database (*n* = 506).

Characteristics	No. of patients	Percentage (%)
Age		
≤65	435	85.97
>65	71	14.03
Gender		
Female	370	73.12
Male	136	26.88
Stage		
I	285	56.32
II	52	10.28
III	112	22.13
IV	55	10.87
Unknown	2	0.40
T category		
T1	144	28.46
T2	167	33.00
T3	170	33.60
T4	23	4.55
Unknown	2	0.40
N category		
N0	230	45.45
N1	226	44.66
Unknown	50	9.88
M category		
M0	283	55.93
M1	9	1.78
Unknown	214	42.29

### Survival-Associated AS Prognostic Signature Construction

**Figure 3:**
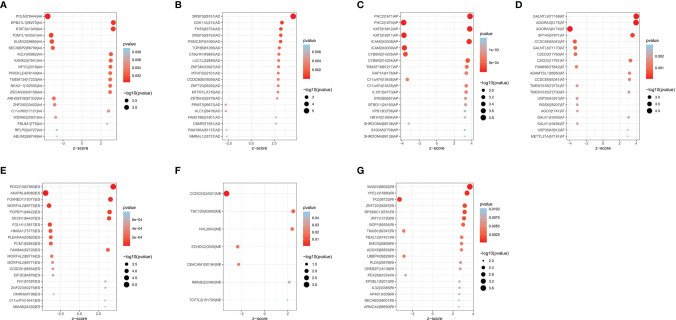
Bubble plots of top 20 significantly OS-related AS events in THCA. Seven types of AS events were displayed from **(A–G)**.

**Figure 4:**
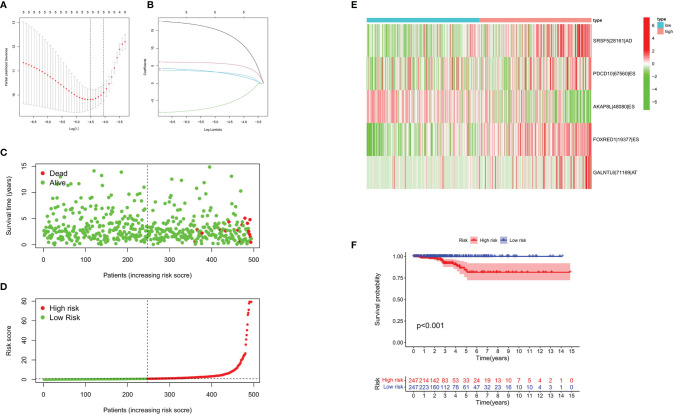
Construction of prognostic signature based on AS events. **(A)** LASSO coefficient of selected OS-related AS events. **(B)** Lambda graph of the LASSO regression signature. **(C, D)** Survival time and survival status of patients in the high- and the low-risk groups. **(E)** Heat map showed the differential expression of five AS events between the high- and the low-risk groups. **(F)** K-M Survival curve of patients in the high- and the low-risk groups.

A total of 1,089 AS events in 823 genes associated with THCA progression were identified by univariate Cox regression analysis with a filter of *p* < 0.05 (**Figures 2D, E**). The distribution of OS-related AS events was shown in the volcano plot (**Figure 2F**). The top 20 OS-related seven types of alternative splicing events are shown in **Figures 3A**–**G**. To avoid overfitting, LASSO regression was employed to determine the final OS-related AS events that were highly associated with THCA (**Figures 4A, B**). Independent prognostic AS events were further identified by multivariate Cox regression. As a result, five AS events, SRSF5-28161-AD, PDCD10-67560-ES, AKAP8L-48080-ES, GALNTL6-71169-AT, and FOXRED1-19377-ES were selected as independent risk factors for constructing the prognostic signature in THCA. The detail of each AS event was recorded (**Supplementary Table S1**).

### Prognostic Signature Validation

**Figure 5:**
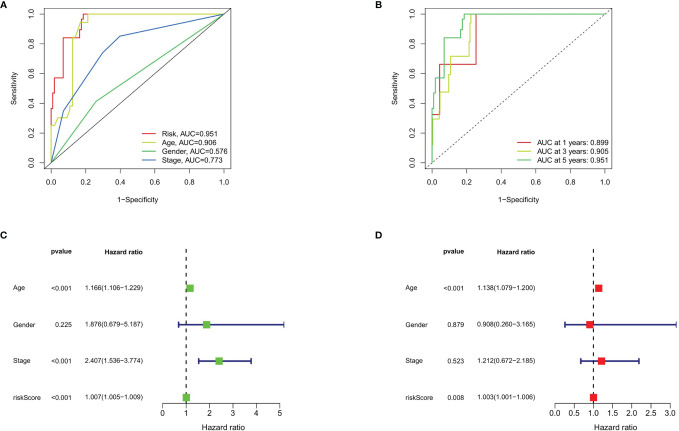
Prognostic signature validation. **(A)** The ROC curves of risk score, age, gender, and stage in THCA. **(B)** The ROC curves of prognostic signature in THCA at 1, 3, and 5 years. **(C)** Forest plot of risk score and clinical data analyzed by univariate Cox regression. **(D)** Univariate Cox regression based on risk score and clinical data analyzed by univariate Cox regression.

**Figure 6:**
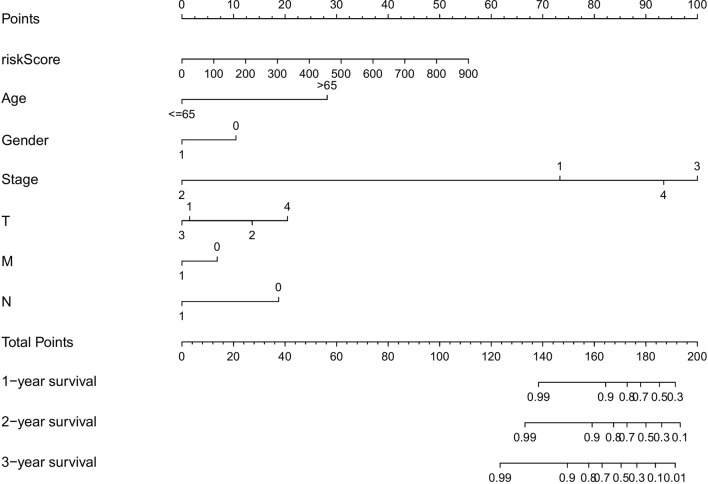
The nomograph, based on clinical data and risk score, was created to predict the 1-, 2-, and 3-year survival rate of THCA patients.

To evaluate the predictive efficiency of the prognostic signature, heat map, risk score plots, K-M curve, and ROC curve were drawn. The risk score distribution curve showed that the higher the risk score of the THCA patients, the shorter is the corresponding survival time (**Figures 4C, D**). The heat map exhibited the expression of five DEASs in patients of the high- and low-risk groups (**Figure 4E**). K-M survival curve showed that the THCA patients in the high-risk group tend to have shorter OS, *p* < 0.001 (**Figure 4F**). Clinical feature-dependent ROC curve showed that the AUC of risk score, age, gender, and stage were 0.951, 0.906, 0.576, and 0.773, respectively (**Figure 5A**). Time-dependent ROC curve showed that the AUC value of 1, 3, and 5 years was 0.899, 0.905, and 0.951, respectively (**Figure 5B**). The HR values for OS calculated by univariate and multivariate Cox regression analyses were 1.007 and 1.003, respectively (*p* < 0.001 and *p* = 0.008, **Figures 5C, D**). These data indicated that the AS-related prognostic signature could be applied to predict OS of patients with THCA. Meanwhile, we constructed a nomogram model to predict the 1-, 2-, and 3-year survival rates of the THCA patients (**Figure 6**).

### Relationship Between Immune Microenvironment and Prognostic Signature

**Figure 7:**
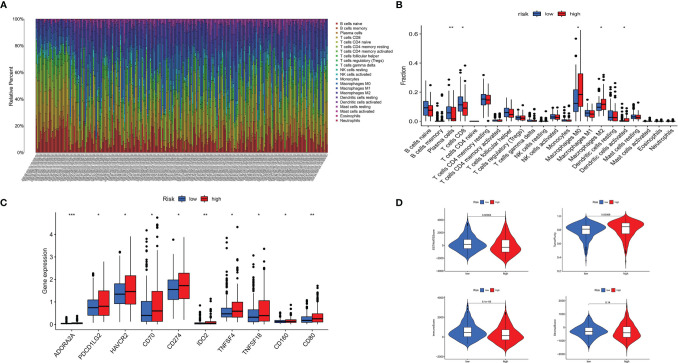
Immune landscape of patients with THCA. **(A)** Proportion of 22 types of immune cells in 495 samples. **(B)** twenty-two types of infiltrating immune cells between the high- and low-risk groups in THCA. **(C)** Immune score between the high- and the low-risk groups in THCA. **(D)** Immune checkpoints between the high- and low-risk groups in THCA. ^*^ *p* < 0.05, ^**^ *p* < 0.01, ^***^ *p* < 0.001.

CIBERSORT was applied to identify the 22 types of immune cells. The proportion of infiltrated immune cells in THCA is shown in **Figure 7A**. Macrophage (M2) cells and CD8+ T cell were the main immune cell types that infiltrated the thyroid cancer tissue among all identified immune cells. Patients in the high-risk group exhibited lower proportion of plasma cells and CD8+ T cells compared with that in the low-risk group. However, the proportion of dendritic cells and macrophages (M0 and M2) were higher in patients of the low-risk group (**Figure 7B**). The above result was consistent with the correlation between immune cells and risk score. The heatmap showed the difference of immune cells and immune response between the high- and low-risk groups for each of the THCA sample (**Supplementary Figure S1**). We found that the proportion of most immune cells was higher in the high-risk group except for macrophages, and most immune response was more active in the low-risk group (**Supplementary Figure S2**).

### Relationship Between Immune Checkpoint Genes, Immune Scores, and Prognostic Signature

Patients in the high-risk group displayed higher expression of immune checkpoints, including ADORA2A, PDCD1LG2 (PD-L2), HAVCR2, CD70, IDO2, TNFSF4, TNFSF18, CD160, CD80, and CD274 (PD-L1), compared with that in the low-risk group (**Figure 7C**). The stromal score, ESTIMATE score, and immune score were higher in the low-risk group, while the result of tumor purity was the opposite (**Figure 7D**).

### Regulatory Network of DEASs and SFs

**Figure 8:**
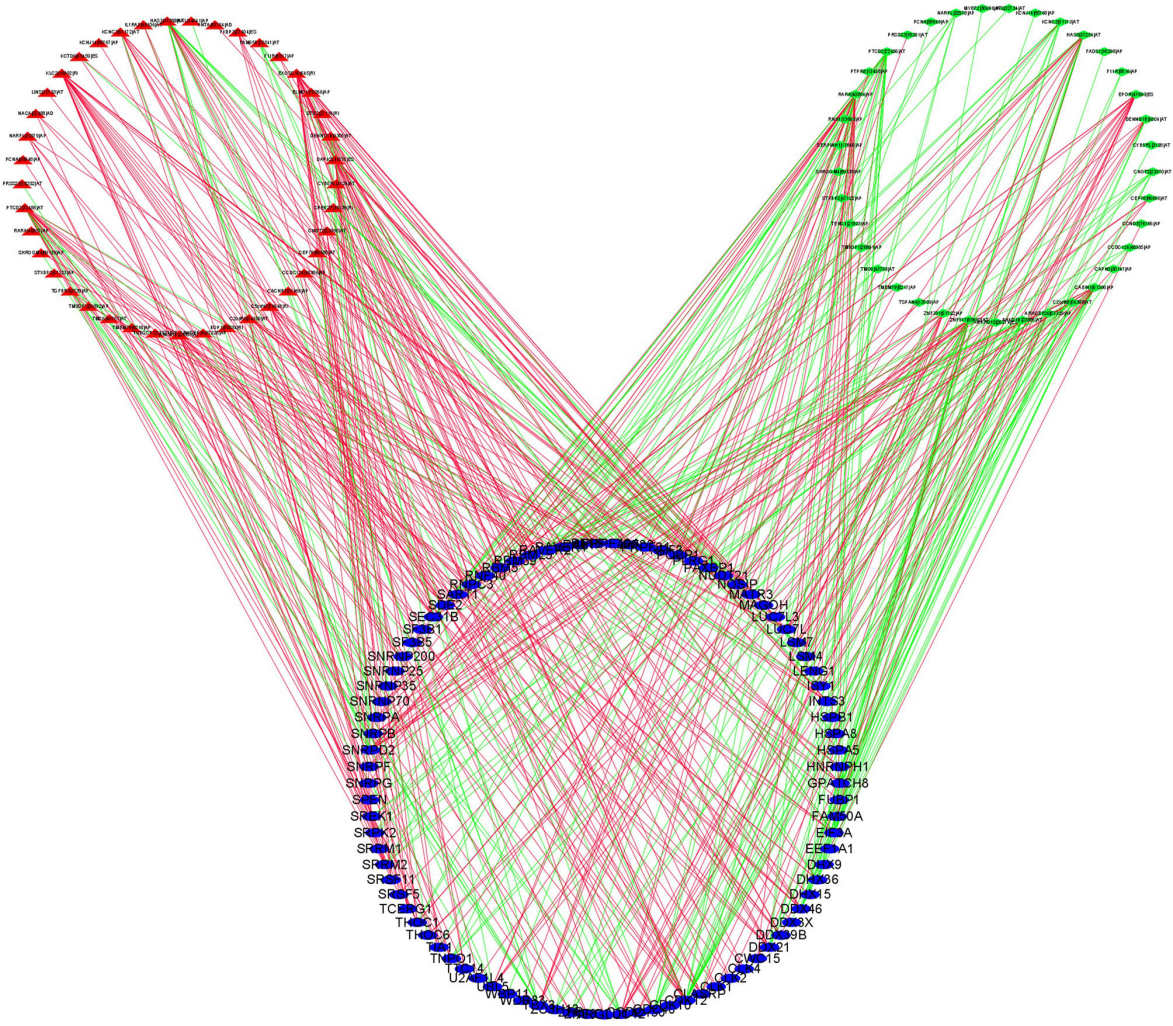
SF-DEAS regulatory network in THCA. Forty-one OS-related high-risk AS events (red triangles) and 37 OS-related low-risk AS events (green diamonds) were positively (green lines) or negatively (red lines) regulated by 89 SFs (blue ellipses).

In order to explore the potential regulatory relationship between the DEASs and SFs, the correlation between the PSI value of prognostic-related DEASs and the expression of SFs was analyzed in the THCA. A total of 78 DEAS events, including 41 high-risk and 37 low-risk AS events, were significantly correlated with 89 SFs (absolute value of *R* ≥ 0.6 and adjusted *p* < 0.001) (**Figure 8**). In the regulatory networks of SFs and DEAS, their relationship was not a simple one. A DEAS can be regulated by up to 42 different SFs, and one SF can regulate up to 24 DEASs. These data indicated the comprehensive regulatory network of cooperation or competition between DEASs and SFs.

## Discussion

Recent studies have focused on the effects of AS events in cancer progression and recurrence. The recurrence of AS events can exert an impact on cancer prognosis (15). Previous studies indicated that abnormal AS events can lead to various diseases, especially cancers, but the mechanism is unclear (16). The AS events play an indispensable role in the progression and metastasis of THCA, which has been proven in many studies (11, 17, 18). However, due to the limited sample size and focusing on specific AS events, the previous research on AS events in THCA was not comprehensive. Therefore, we made a comprehensive analysis of AS events in a large sample size of THCA in order to clarify the mechanism of AS events in THCA.

In this study, we detected 45,150 AS events in 21,075 genes in 495 THCA patients, suggesting that AS was a ubiquitous biological process in THCA and mainly involved in the regulation of posttranscriptional modification. In addition, 1,089 DEASs were identified from the comparison between THCA and normal tissues. Previous study found that most DEASs were closely related to their corresponding genes, which indicated that AS plays an indispensable role in the posttranscriptional process and can change gene expression (19). Network of prognostic-related DEASs and SFs further clarified potential pathways associated with AS events. All the important splicing variants verified in previous THCA studies have also been identified by our program, which showed that the results we found were reliable and the DEASs found in this study were common in THCA. At the same time, we also found that some common DEASs were detected in THCA, colorectal cancer, and head and neck squamous cell carcinoma, indicating that some AS events were ubiquitous in the occurrence and development in different types of cancers (20, 21).

SRSF5, PDCD10, AKAP8L, FOXRED1, and GALNTL6 were finally identified as integrated prognostic splicing biomarkers in our study. The prognosis of patients with THCA can be predicted accurately predicted by the signature composed with five AS events. According to NCBI, Ensemble, and SMART database, SRSF5 encoded the protein which was an important constituent element of pre-mRNA splicing factors and forms part of the spliceosome (22). Previous studies have found that SRSF5 can regulate the m6A methylation of pancreatic cancer, thus promoting its growth and metastasis (23). SRSF5 has been investigated as a novel oncogene that is involved in oral squamous cell carcinoma and can be upregulated by SRSF3 (24). PDCD10 was one of the CCM families of proteins and also known as cerebral cavernous malformations 3 (CCM3) (25, 26). PDCD10 overexpression can accelerate tumor migration, invasion through reversing TRIM59 loss-induced contractile phenotypes (27). AKAP8L was found to regulate translation, cell growth, and cell proliferation (28). FOXRED1 encodes a protein that contains a FAD-dependent oxidoreductase domain and has been investigated as a new biomarker in human colorectal cancer (29). Passon found that amplifications of GALNTL6 were more likely to be identified in the high-risk group than that of the low-risk group in THCA (30). We found that all the above five genes were widely involved in the biological process of cancers. Therefore, we assumed that the changes in these genes may be related to the formation and progression of cancers and that the mechanism of related splicing events should be further identified.

Immunotherapy targeting immune checkpoints has been proven to improve the prognosis of patients with THCA. Therefore, the difference of immune checkpoint expression between the high- and low-risk groups was analyzed. The results of our study indicated that the patients in the high-risk group had higher expressions of immune checkpoint proteins (ADORA2A, PDCD1LG2, HAVCR2, CD70, IDO2, TNFSF4, TNFSF18, CD160, CD80, and CD274). An increasing body of evidence showed that CD274 (PD-L1) played a key role in the TME of various tumors. Nikita and Yoo found that high expression of PD-L1 was associated with poor clinical outcomes for THCA patients (31, 32). However, the expression of another PD-1 ligand, PCCDLG2 (PD-L2), has not been fully elucidated, especially in thyroid cancer. PD-L2 expression can be detected in various immune cells and tumor cells and is easily affected by the change of microenvironment (33–35). Daisuke’s research confirmed that CD80 limits the PD-1 coinhibitory signal and induces T-cell proliferation and cytokine production (36). HAVCR2 (Tim-3) plays an important role in inhibiting the expression of cytokines such as TNF and INF-γ that are cytokines widely existing in many immune response (37).

Cancer cells, endothelial cells, and tumor-infiltrating lymphocytes are the main cells that express HARVR2 (38). It has been proven that the expression of HAVCR2 were significantly higher in tumor tissue samples compared with paracarcinoma tissue. Meanwhile, high expression of HAVCR2 was significantly correlated with the poor prognosis of ovarian cancer (39), colon cancer (40), bladder urothelial carcinoma (41), and gastric cancer (42). Lawrence et al. performed a study of an A2AR antagonist for cancer treatment and demonstrated the antitumor activity in patients with refractory renal cell cancer (43). Several new immune checkpoint proteins that have not been previously reported in thyroid cancer have been found. Therefore, the differentially expressed immune checkpoint proteins between patients with high- and low-risk scores may provide a new solution for immunotherapy of THCA.

It has been reported that AS events are widely involved in angiogenesis, invasion, and immune destruction of a variety of tumors (44). New epitopes, produced by gene-derived AS events, can be used in immunotherapy to improve patient survival rate. However, at present, there were few studies on the bioinformatics analysis of AS and immunity in THCA (45). In this study, we described the relationship between AS events and immunity and revealed the different distributions of immune cells in the high- and low-risk groups. The differential distribution of immune cells, such as plasma cells, CD8+ T cells, macrophages (M0), macrophages (M2), and dendritic cells was identified. Among them, low proportion of plasma cells and CD8+ T cells was clearly correlated with poor prognosis, while high proportion of macrophages (M0), macrophages (M2), and dendritic cells was correlated with better prognosis. A similar trend has been identified in the study of skin cancer (46) and malignant melanoma (47). In brief, our research showed that there was a strong correlation between AS events and immune cell infiltration in THCA.

Immunotherapy, which was based on immune cells, can provide a new insight for the treatment of some cancers, including THCA (48, 49). However, the problem faced by researchers was how to find a suitable target antigen for immunotherapy. In the process of searching for new tumor mutant antigens, attention has been paid to the new epitopes produced by mRNA AS events. Recent studies have found that peptides produced by mRNA AS events may combine with MHC-I molecules to produce complexes that can act as new epitopes (49). Therefore, assuming that the AS events identified in the present study can discover new epitopes for CD8+ T cells, plasma cells, or other immune cells, the alternative targets for cancer immunotherapy will be greatly expanded. As for clinical transformation, personalized vaccines prepared with new antigenic peptides can produce T-cell responses *in vivo*, which can reduce or eliminate tumor cells and improve the prognosis of patients with cancers.

## Conclusions

Taken together, a prognostic signature consisting of five AS events in THCA was established, which was helpful for individualized and accurate treatment of patients. Furthermore, there was an inextricable correlation between immune cell infiltration, immune checkpoints, and AS events. This result is of great significance and provides as basis for THCA immunotherapy in the future.

## Data Availability Statement

The original contributions presented in the study are included in the article/**Supplementary Material**. Further inquiries can be directed to the corresponding authors.

## Ethics Statement

Ethical review and approval was not required for the study on human participants in accordance with the local legislation and institutional requirements. Written informed consent for participation was not required for this study in accordance with the national legislation and the institutional requirements.

## Author Contributions

The original draft was prepared and written by JW and YS. JL, MA, and LY were in charge of the data curation. LY reviewed and edited the article. JS and FY were in charge of the project administration. All authors commented and approved the text. All authors contributed to the article and approved the submitted version.

## Funding

This work was supported by the Natural Science Foundation of Guangdong province (No. 2018B0303110013).

## Conflict of Interest

The authors declare that the research was conducted in the absence of any commercial or financial relationships that could be construed as a potential conflict of interest.

## Publisher’s Note

All claims expressed in this article are solely those of the authors and do not necessarily represent those of their affiliated organizations, or those of the publisher, the editors and the reviewers. Any product that may be evaluated in this article, or claim that may be made by its manufacturer, is not guaranteed or endorsed by the publisher.

## Supplementary Material

Supplementary Figure 1 The correlation of differential infiltrated immune cells and risk score in THCA. **(A)** The correlation of CD8+ T cell and risk score. **(B)** The correlation of plasma cells and risk score. **(C)** The correlation of dendritic cell and risk score. **(D)** The correlation of macrophages (M0) and risk score. **(E)** The correlation of macrophages (M2) and risk score.

Supplementary Figure 2 Heat map showed the differential immune response and infiltration of immune cells between the high- and the low-risk groups in THCA.

## References

[1] Davies L Hoang JK. Thyroid Cancer in the USA: Current Trends and Outstanding Questions. Lancet Diabetes Endocrinol (2021) 9(1):11–2. doi: 10.1016/S2213-8587(20)30372-7 33220765

[2] Nikiforova MN Tseng GC Steward D Diorio D Nikiforov YE. MicroRNA Expression Profiling of Thyroid Tumors: Biological Significance and Diagnostic Utility. J Clin Endocrinol Metab (2008) 93(5):1600–8. doi: 10.1210/jc.2007-2696 PMC2386678 18270258

[3] Lloyd RV Buehler D Khanafshar E. Papillary Thyroid Carcinoma Variants. Head Neck Pathol (2011) 5(1):51–6. doi: 10.1007/s12105-010-0236-9 PMC3037461 21221869

[4] Colombo C Giancola N Fugazzola L. Personalized Treatment for Differentiated Thyroid Cancer: Current Data and New Perspectives. Minerva Endocrinol (Torino) (2021) 46(1):62–89. doi: 10.23736/S0391-1977.20.03342-8 33213119

[5] Feng H Qin Z Zhang X. Opportunities and Methods for Studying Alternative Splicing in Cancer With RNA-Seq. Cancer Lett (2013) 340(2):179–91. doi: 10.1016/j.canlet.2012.11.010 23196057

[6] Wan L Yu W Shen E Sun W Liu Y Kong J. SRSF6-Regulated Alternative Splicing That Promotes Tumour Progression Offers a Therapy Target for Colorectal Cancer. Gut (2019) 68(1):118–29. doi: 10.1136/gutjnl-2017-314983 29114070

[7] Oltean S Bates DO. Hallmarks of Alternative Splicing in Cancer. Oncogene (2014) 33(46):5311–8. doi: 10.1038/onc.2013.533 24336324

[8] Yao J Caballero OL Huang Y Lin C Rimoldi D Behren A. Altered Expression and Splicing of ESRP1 in Malignant Melanoma Correlates With Epithelial-Mesenchymal Status and Tumor-Associated Immune Cytolytic Activity. Cancer Immunol Res (2016) 4(6):552–61. doi: 10.1158/2326-6066.CIR-15-0255 27045022

[9] Qi F Li Y Yang X Wu YP Lin LJ Liu XM. Significance of Alternative Splicing in Cancer Cells. Chin Med J (Engl) (2020) 133(2):221–8. doi: 10.1097/CM9.0000000000000542 PMC7028187 31764175

[10] Lazzereschi D Nardi F Turco A Ottini L D'Amico C Mariani-Costantini R. A Complex Pattern of Mutations and Abnormal Splicing of Smad4 is Present in Thyroid Tumours. Oncogene (2005) 24(34):5344–54. doi: 10.1038/sj.onc.1208603 15940269

[11] Montero-Conde C Grana-Castro O Martin-Serrano G Martinez-Montes AM Zarzuela E Munoz J. Hsa-miR-139-5p is a Prognostic Thyroid Cancer Marker Involved in HNRNPF-Mediated Alternative Splicing. Int J Cancer (2020) 146(2):521–30. doi: 10.1002/ijc.32622 31403184

[12] Ryan MC Cleland J Kim R Wong WC Weinstein JN. SpliceSeq: A Resource for Analysis and Visualization of RNA-Seq Data on Alternative Splicing and its Functional Impacts. Bioinformatics (2012) 28(18):2385–7. doi: 10.1093/bioinformatics/bts452 PMC3436850 22820202

[13] Liberzon A Birger C Thorvaldsdottir H Ghandi M Mesirov JP Tamayo P. The Molecular Signatures Database (MSigDB) Hallmark Gene Set Collection. Cell Syst (2015) 1(6):417–25. doi: 10.1016/j.cels.2015.12.004 PMC4707969 26771021

[14] Shannon P Markiel A Ozier O Baliga NS Wang JT Ramage D. Cytoscape: A Software Environment for Integrated Models of Biomolecular Interaction Networks. Genome Res (2003) 13(11):2498–504. doi: 10.1101/gr.1239303 PMC403769 14597658

[15] Shkreta L Chabot B. The RNA Splicing Response to DNA Damage. Biomolecules (2015) 5(4):2935–77. doi: 10.3390/biom5042935 PMC4693264 26529031

[16] Cahill K. Alternative Splicing and Genomic Stability. Phys Biol (2004) 1(1-2):C1–4. doi: 10.1088/1478-3967/1/2/C01 16204811

[17] Wu ZH Tang Y Zhou Y. Alternative Splicing Events Implicated in Carcinogenesis and Prognosis of Thyroid Gland Cancer. Sci Rep (2021) 11(1):4841. doi: 10.1038/s41598-021-84403-6 33649373 PMC7921437

[18] Lin P He RQ Huang ZG Zhang R Wu HY Shi L. Role of Global Aberrant Alternative Splicing Events in Papillary Thyroid Cancer Prognosis. Aging (Albany NY) (2019) 11(7):2082–97. doi: 10.18632/aging.101902 PMC6503875 30986203

[19] Zhou R Moshgabadi N Adams KL. Extensive Changes to Alternative Splicing Patterns Following Allopolyploidy in Natural and Resynthesized Polyploids. Proc Natl Acad Sci USA (2011) 108(38):16122–7. doi: 10.1073/pnas.1109551108 PMC3179116 21900601

[20] Xiong Y Deng Y Wang K Zhou H Zheng X Si L. Profiles of Alternative Splicing in Colorectal Cancer and Their Clinical Significance: A Study Based on Large-Scale Sequencing Data. Ebiomedicine (2018) 36:183–95. doi: 10.1016/j.ebiom.2018.09.021 PMC6197784 30243491

[21] Li ZX Zheng ZQ Wei ZH Zhang LL Li F Lin L. Comprehensive Characterization of the Alternative Splicing Landscape in Head and Neck Squamous Cell Carcinoma Reveals Novel Events Associated With Tumorigenesis and the Immune Microenvironment. Theranostics (2019) 9(25):7648–65. doi: 10.7150/thno.36585 PMC6831462 31695792

[22] Chen Y Huang Q Liu W Zhu Q Cui CP Xu L. Mutually Exclusive Acetylation and Ubiquitylation of the Splicing Factor SRSF5 Control Tumor Growth. Nat Commun (2018) 9(1):2464. doi: 10.1038/s41467-018-04815-3 29942010 PMC6018636

[23] Chen S Yang C Wang ZW Hu JF Pan JJ Liao CY. CLK1/SRSF5 Pathway Induces Aberrant Exon Skipping of METTL14 and Cyclin L2 and Promotes Growth and Metastasis of Pancreatic Cancer. J Hematol Oncol (2021) 14(1):60. doi: 10.1186/s13045-021-01072-8 33849617 PMC8045197

[24] Yang S Jia R Bian Z. SRSF5 Functions as a Novel Oncogenic Splicing Factor and is Upregulated by Oncogene SRSF3 in Oral Squamous Cell Carcinoma. Biochim Biophys Acta Mol Cell Res (2018) 1865(9):1161–72. doi: 10.1016/j.bbamcr.2018.05.017 29857020

[25] Zhou Z Rawnsley DR Goddard LM Pan W Cao XJ Jakus Z. The Cerebral Cavernous Malformation Pathway Controls Cardiac Development via Regulation of Endocardial MEKK3 Signaling and KLF Expression. Dev Cell (2015) 32(2):168–80. doi: 10.1016/j.devcel.2014.12.009 PMC4589864 25625206

[26] Chen PY Chang WS Lai YK Wu CW. C-Myc Regulates the Coordinated Transcription of Brain Disease-Related PDCD10-SERPINI1 Bidirectional Gene Pair. Mol Cell Neurosci (2009) 42(1):23–32. doi: 10.1016/j.mcn.2009.05.001 19442737

[27] Tan P He L Zhou Y. TRIM59 Deficiency Curtails Breast Cancer Metastasis Through SQSTM1-Selective Autophagic Degradation of PDCD10. Autophagy (2019) 15(4):747–9. doi: 10.1080/15548627.2019.1569951 PMC6526857 30653426

[28] Melick CH Meng D. Jewell JL. A-Kinase Anchoring Protein 8L Interacts With Mtorc1 and Promotes Cell Growth. J Biol Chem (2020) 295(23):8096–105. doi: 10.1074/jbc.AC120.012595 PMC7278349 32312749

[29] Fei W Liu S Hu X. High FOXRED1 Expression Predicted Good Prognosis of Colorectal Cancer. Am J Cancer Res (2016) 6(11):2722–8. PMC5126286 27904784

[30] Passon N Bregant E Sponziello M Dima M Rosignolo F Durante C. Somatic Amplifications and Deletions in Genome of Papillary Thyroid Carcinomas. Endocrine (2015) 50(2):453–64. doi: 10.1007/s12020-015-0592-z 25863487

[31] Pozdeyev N Gay LM Sokol ES Hartmaier R Deaver KE Davis S. Genetic Analysis of 779 Advanced Differentiated and Anaplastic Thyroid Cancers. Clin Cancer Res (2018) 24(13):3059–68. doi: 10.1158/1078-0432.CCR-18-0373 PMC6030480 29615459

[32] Yoo SK Song YS Lee EK Hwang J Kim HH Jung G. Integrative Analysis of Genomic and Transcriptomic Characteristics Associated With Progression of Aggressive Thyroid Cancer. Nat Commun (2019) 10(1):2764. doi: 10.1038/s41467-019-10680-5 31235699 PMC6591357

[33] Okazaki T Honjo T. PD-1 and PD-1 Ligands: From Discovery to Clinical Application. Int Immunol (2007) 19(7):813–24. doi: 10.1093/intimm/dxm057 17606980

[34] Rozali EN Hato SV Robinson BW Lake RA Lesterhuis WJ. Programmed Death Ligand 2 in Cancer-Induced Immune Suppression. Clin Dev Immunol (2012) 2012:656340. doi: 10.1155/2012/656340 22611421 PMC3350956

[35] Zhong X Tumang JR Gao W Bai C Rothstein TL. PD-L2 Expression Extends Beyond Dendritic Cells/Macrophages to B1 Cells Enriched for V(H)11/V(H)12 and Phosphatidylcholine Binding. Eur J Immunol (2007) 37(9):2405–10. doi: 10.1002/eji.200737461 17683117

[36] Sugiura D Maruhashi T Okazaki IM Shimizu K Maeda TK Takemoto T. Restriction of PD-1 Function by Cis-PD-L1/CD80 Interactions is Required for Optimal T Cell Responses. Science (2019) 364(6440):558–66. doi: 10.1126/science.aav7062 31000591

[37] Wu W Shi Y Li J Chen F Chen Z Zheng M. Tim-3 Expression on Peripheral T Cell Subsets Correlates With Disease Progression in Hepatitis B Infection. Virol J (2011) 8:113. doi: 10.1186/1743-422X-8-113 21392402 PMC3061941

[38] Sakuishi K Apetoh L Sullivan JM Blazar BR Kuchroo VK Anderson AC. Targeting Tim-3 and PD-1 Pathways to Reverse T Cell Exhaustion and Restore Anti-Tumor Immunity. J Exp Med (2010) 207(10):2187–94. doi: 10.1084/jem.20100643 PMC2947065 20819927

[39] Sawada M Goto K Morimoto-Okazawa A Haruna M Yamamoto K Yamamoto Y. PD-1+ Tim3+ Tumor-Infiltrating CD8 T Cells Sustain the Potential for IFN-Gamma Production, But Lose Cytotoxic Activity in Ovarian Cancer. Int Immunol (2020) 32(6):397–405. doi: 10.1093/intimm/dxaa010 32009163

[40] Zhou E Huang Q Wang J Fang C Yang L Zhu M. Up-Regulation of Tim-3 is Associated With Poor Prognosis of Patients With Colon Cancer. Int J Clin Exp Pathol (2015) 8(7):8018–27. PMC4555696 26339368

[41] Yang M Yu Q Liu J Fu W Cao Y Yu L. T-Cell Immunoglobulin Mucin-3 Expression in Bladder Urothelial Carcinoma: Clinicopathologic Correlations and Association With Survival. J Surg Oncol (2015) 112(4):430–5. doi: 10.1002/jso.24012 26265374

[42] Jiang J Jin MS Kong F Cao D Ma HX Jia Z. Decreased Galectin-9 and Increased Tim-3 Expression are Related to Poor Prognosis in Gastric Cancer. PloS One (2013) 8(12):e81799. doi: 10.1371/journal.pone.0081799 24339967 PMC3858245

[43] Fong L Hotson A Powderly JD Sznol M Heist RS Choueiri TK. Adenosine 2a Receptor Blockade as an Immunotherapy for Treatment-Refractory Renal Cell Cancer. Cancer Discov (2020) 10(1):40–53. doi: 10.1158/2159-8290.CD-19-0980 31732494 PMC6954326

[44] Lee SC Abdel-Wahab O. Therapeutic Targeting of Splicing in Cancer. Nat Med (2016) 22(9):976–86. doi: 10.1038/nm.4165 PMC5644489 27603132

[45] Frankiw L Baltimore D Li G. Alternative mRNA Splicing in Cancer Immunotherapy. Nat Rev Immunol (2019) 19(11):675–87. doi: 10.1038/s41577-019-0195-7 31363190

[46] Beer TW Ng LB Murray K. Mast Cells Have Prognostic Value in Merkel Cell Carcinoma. Am J Dermatopathol (2008) 30(1):27–30. doi: 10.1097/DAD.0b013e31815c932a 18212540

[47] Ribatti D Ennas MG Vacca A Ferreli F Nico B Orru S. Tumor Vascularity and Tryptase-Positive Mast Cells Correlate With a Poor Prognosis in Melanoma. Eur J Clin Invest (2003) 33(5):420–5. doi: 10.1046/j.1365-2362.2003.01152.x 12760367

[48] Paucek RD Baltimore D Li G. The Cellular Immunotherapy Revolution: Arming the Immune System for Precision Therapy. Trends Immunol (2019) 40(4):292–309. doi: 10.1016/j.it.2019.02.002 30871979

[49] Jayasinghe RG Cao S Gao Q Wendl MC Vo NS Reynolds SM. Systematic Analysis of Splice-Site-Creating Mutations in Cancer. Cell Rep (2018) 23(1):270–81. doi: 10.1016/j.celrep.2018.03.052 PMC6055527 29617666

