# Supplementary figures and images for: Analysis of Prognostic Alternative Splicing Reveals the Landscape of Immune Microenvironment in Thyroid Cancer
Source: Front Oncol. 2021 Oct 18;11:763886. doi: 10.3389/fonc.2021.763886 (PMC8558422; doi:10.3389/fonc.2021.763886)

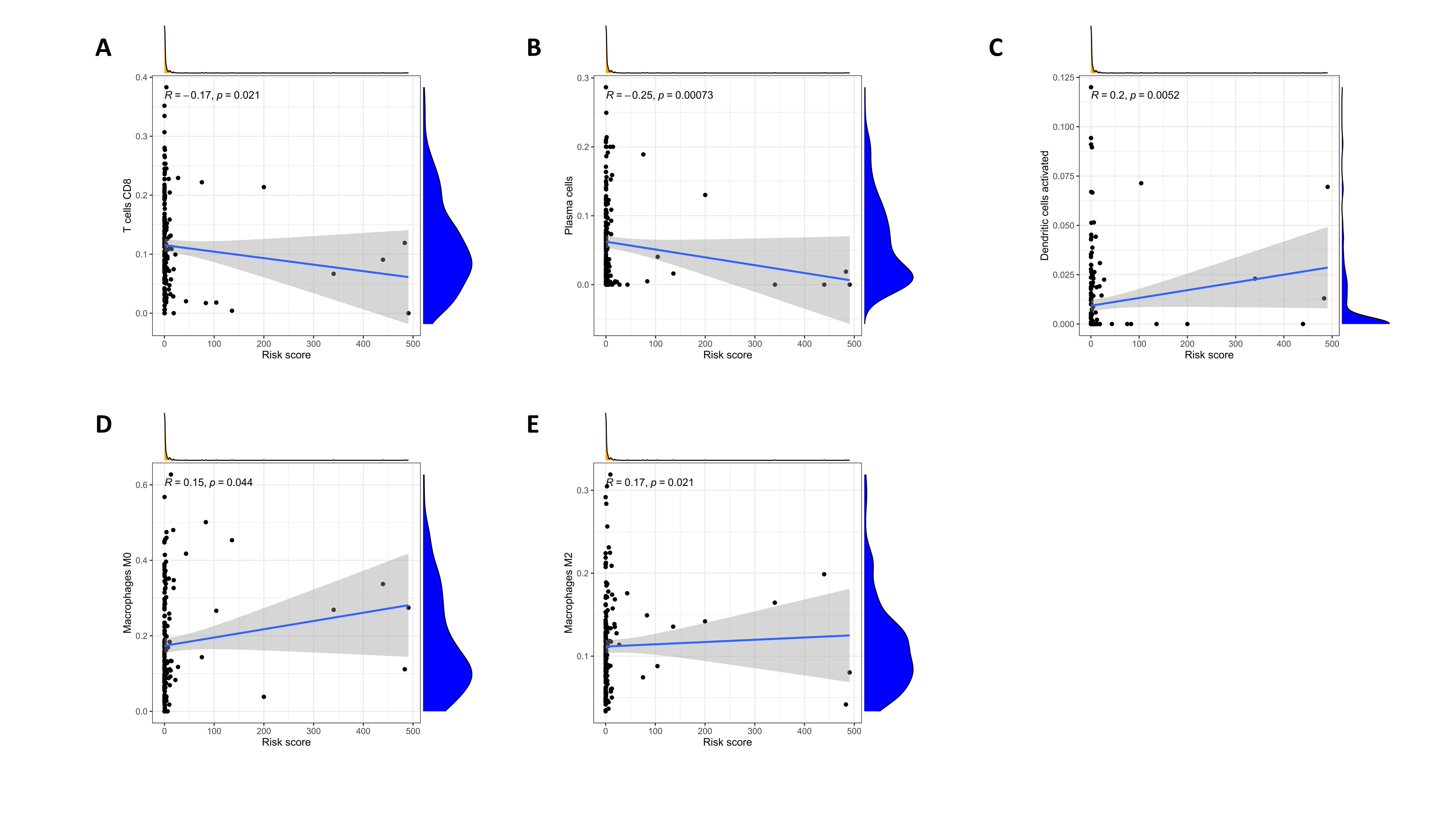

Supplement: Supplementary Figure 1 — The correlation of differential infiltrated immune cells and risk score in THCA. (A) The correlation of CD8+ T cell and risk score. (B) The correlation of plasma cells and risk score. (C) The correlation of dendritic cell and risk score. (D) The correlation of macrophages (M0) and risk score. (E) The correlation of macrophages (M2) and risk score. [file Image_1.jpeg]

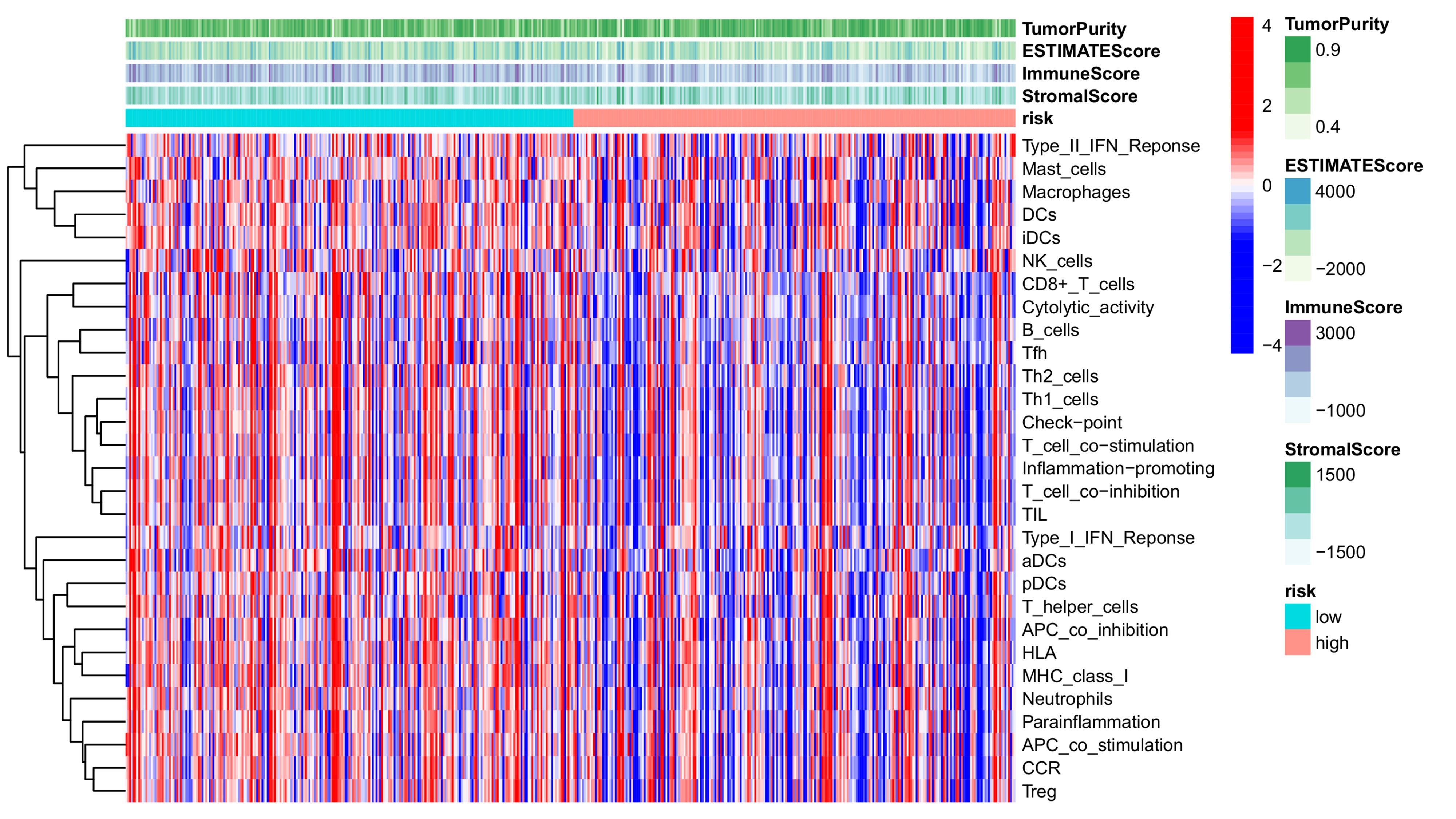

Supplement: Supplementary Figure 2 — Heat map showed the differential immune response and infiltration of immune cells between the high- and the low-risk groups in THCA. [file Image_2.jpeg]
