# Supplementary material for: Analysis of Prognostic Alternative Splicing Reveals the Landscape of Immune Microenvironment in Thyroid Cancer
Source: Front Oncol. 2021 Oct 18;11:763886. doi: 10.3389/fonc.2021.763886 (PMC8558422; doi:10.3389/fonc.2021.763886)
Supplement: Supplementary file 3 [file Table_1.docx]

Table S1 Multivariate cox mode

| **ID** | **coef** | **HR** | **HR.95L** | **HR.95H** | **pvalue** |
| --- | --- | --- | --- | --- | --- |
| SRSF5\|28161\|AD | 18.060 | 69754264.586 | 9517.678 | 5.11223E+11 | 6.96E-05 |
| PDCD10\|67560\|ES | 6.120 | 454.945 | 4.759 | 43489.239 | 0.009 |
| AKAP8L\|48080\|ES | -8.653 | 0.000 | 1.63E-06 | 0.019 | 0.000 |
| FOXRED1\|19377\|ES | 4.449 | 85.553 | 2.800 | 2614.457 | 0.011 |
| GALNTL6\|71169\|AT | 3.813 | 45.273 | 1.206 | 1699.670 | 0.039 |
